# Hippocampal Sequences During Exploration: Mechanisms and Functions

**DOI:** 10.3389/fncel.2019.00232

**Published:** 2019-06-13

**Authors:** Céline Drieu, Michaël Zugaro

**Affiliations:** Center for Interdisciplinary Research in Biology, Collège de France, CNRS UMR 7241, INSERM U 1050, PSL Research University, Paris, France

**Keywords:** place cell, network dynamics, phase precession, theta sequences, awake replay, memory

## Abstract

Although the hippocampus plays a critical role in spatial and episodic memories, the mechanisms underlying memory formation, stabilization, and recall for adaptive behavior remain relatively unknown. During exploration, within single cycles of the ongoing theta rhythm that dominates hippocampal local field potentials, place cells form precisely ordered sequences of activity. These neural sequences result from the integration of both external inputs conveying sensory-motor information, and intrinsic network dynamics possibly related to memory processes. Their endogenous replay during subsequent sleep is critical for memory consolidation. The present review discusses possible mechanisms and functions of hippocampal theta sequences during exploration. We present several lines of evidence suggesting that these neural sequences play a key role in information processing and support the formation of initial memory traces, and discuss potential functional distinctions between neural sequences emerging during theta vs. awake sharp-wave ripples.

## 1. Introduction

The ability of brain circuits to generate precise sequences of neuronal activity may underlie certain forms of sensory-motor processing and learning (Skaggs et al., 1996; Riehle et al., 1997; Stopfer et al., 1997; Petersen et al., 2001). While in some systems these sequences may be driven by external stimuli or motor signals, reports of internally generated neuronal sequences in the hippocampus, under either minimal (e.g., sleep; Lee and Wilson, 2002; Ji and Wilson, 2007) or invariant (Pastalkova et al., 2008; Wang et al., 2015) sensory-motor inputs, have identified a potential evolutionary mechanism for planning and memory consolidation (Lisman and Redish, 2009; Foster and Knierim, 2012; Buzsáki and Moser, 2013; Wikenheiser and Redish, 2015a; Zielinski et al., 2017).

The hippocampal network receives highly integrated inputs from distributed areas (Witter et al., 1989; Andersen et al., 2007; van Strien et al., 2009) and is endowed with auto-associative properties (Gilbert and Brushfield, 2009), which allows it to integrate and retrieve spatial as well as non-spatial information (Buzsáki and Moser, 2013; Eichenbaum, 2017). Bilateral lesions of the hippocampus result in long term deficits in episodic memory (Scoville and Milner, 1957) as well as in strong impairments in spatial memory (Morris et al., 1982). Indeed, the hippocampal formation has been proposed to support a cognitive map of the environment (Tolman, 1948; O'Keefe and Nadel, 1978), based on the remarkable property of its “place cells” that discharge when the animal occupies restricted locations in the environment (“place fields,” O'Keefe and Dostrovsky, 1971). During the exploration, the animal sequentially crosses successive place fields, and the corresponding place cells fire in sequence, reflecting the ongoing trajectory at the behavioral timescale. Strikingly, embedded in these slow sequences, time-compressed sequences emerge in each cycle of the ongoing theta rhythm (O'Keefe and Recce, 1993; Skaggs et al., 1996), a strong and regular ~8 Hz oscillation of the hippocampal local field potential (LFP; Jouvet, 1969; Vanderwolf, 1969). These sequences are known as “theta sequences.” As a result of this fast timescale organization, the spikes emitted by successive cell assemblies occur within dozens of milliseconds, and this pattern is continually repeated in successive theta cycles, which is conducive to Hebbian synaptic plasticity (Skaggs et al., 1996; Magee and Johnston, 1997; Stuart, 2001; Buzsáki and Draguhn, 2004; Wójtowicz and Mozrzymas, 2015). Therefore, theta sequences have been proposed to allow the formation of spatial and episodic memory traces, by anchoring spatial events in their temporal context (Skaggs et al., 1996; Drieu et al., 2018).

Interestingly, fast time-compressed trajectories are also represented by neuronal sequences that occur during brief pauses and prolonged immobility (Foster and Wilson, 2006; Diba and Buzsáki, 2007), when theta oscillations are absent. Instead, these replay events occur during sharp-wave ripple complexes (O'Keefe, 1976; Buzsáki et al., 1983, 1992), a transient LFP pattern associating fast (~200 Hz) oscillations in *stratum pyramidale* and a large deflection in *stratum radiatum*. These awake replay events have been involved in spatial working memory (Jadhav et al., 2012). Further replay of behavior-related neural sequences endogenously occurs during sleep (Wilson and McNaughton, 1994; Lee and Wilson, 2002), and has been shown to play a critical role in memory consolidation (Girardeau et al., 2009; Ego-Stengel and Wilson, 2010; Maingret et al., 2016; van de Ven et al., 2016).

Hippocampal sequences may thus represent a general neurophysiological mechanism supporting memory formation, consolidation and retrieval. The present review focuses on hippocampal theta sequences. We first describe the cellular and network mechanisms proposed to give rise to the theta-related dynamics of hippocampal place cells. We then report evidence for a role in information processing and memory trace formation. Finally, we discuss possible functional distinctions between neural sequences emerging during theta and awake sharp-wave ripples.

## 2. Emergence of Theta Sequences

The sequential activation of successive place cells along a given trajectory, at the behavioral timescale, does not trivially imply that in overlapping regions of space these same cells should fire in the same order at the much faster, “theta” timescale. How do theta sequences emerge in the hippocampal network? During locomotion, the hippocampal dynamics are under the combined influences of extrinsic inputs from multiple sensory and locomotion-related structures, as well as the intrinsic properties of the hippocampal network, modulated by elevated acetylcholine levels. Theta oscillations dominate the hippocampal LFP and powerfully shape both pyramidal cell and interneuron firing.

### 2.1. Theta During Locomotion

**Figure 1:**
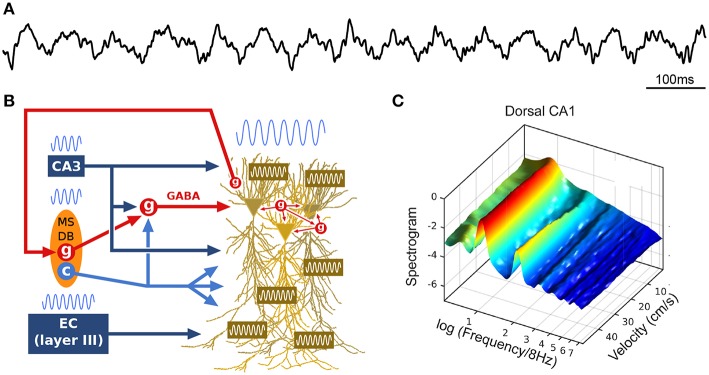
Theta oscillations. **(A)** Example local field potential (LFP) trace during track running. **(B)** Simplified schematic of theta generation. The medial septum-diagonal band of Broca (MSDB) sends theta-rhythmic GABAergic (g), cholinergic (c) and glutamatergic projections to the hippocampus (glutamatergic neurons appear to entrain other septal neurons, but not hippocampal theta, and are not represented here). Rhythmic inhibition (red, GABA) of highly interconnected perisomatic interneurons (g) constrains the timing of pyramidal cell spikes. The entorhinal cortex (EC) also sends theta-paced outputs to the hippocampus through the perforant path. CA3 outputs targeting *stratum radiatum* constitute another likely intrahippocampal theta generator. In addition, the dendritic membrane of CA1 pyramidal cells is endowed with resonant properties at theta frequency. Finally, hippocampal long-range backpropagating interneurons target the MSDB, arguing for cyclical (non-unidirectional) mechanisms in the generation of theta. **(C)** Power spectral density of dorsal CA1 LFP as a function of running velocity (logarithmic scale; average over four rats). At low velocities, power is dominated by a single peak located between 6 and 7 Hz. As velocity increases, the peak quickly narrows, shifts toward 8 Hz, while additional peaks (harmonics) develop at frequencies *n* × 8 Hz (up to *n* = 6 for dorsal CA1 region, *n* = 2 for intermediate CA1). Reproduced from Sheremet et al. (2016).

The theta rhythm includes a range of low and regular frequency oscillations from 4 to 12 Hz (Figure 1A). During wakefulness, theta oscillations arise during locomotion and epochs of active engagement in the environment such as navigating, exploring objects, rearing and preparing for movement (Jouvet, 1969; Vanderwolf, 1969; Foster et al., 1989). While the precise mechanisms responsible for the generation of theta remain to be elucidated (Buzsáki et al., 2002), theta oscillations are thought to result from an interplay between numerous factors (Figure 1B), including rhythmic cholinergic modulation and feed-forward inhibition from the medial septum and diagonal band of Broca (MSDB; Petsche et al., 1962; Kramis et al., 1975; Buzsáki et al., 1986; Vanderwolf et al., 1988; Kocsis et al., 1999; Müller and Remy, 2014; Zutshi et al., 2018), rhythmic inputs from layers II and III of the entorhinal cortex targeting DG/CA3 and CA1, respectively (Buzsáki et al., 1983; Kamondi et al., 1998), activation of CA1 pyramidal cell dendrites by CA3 collaterals (Konopacki et al., 1987; Kamondi et al., 1998; Fisher and Blum, 1999), interneuronal network dynamics within (Goutagny et al., 2009) and between (Jackson et al., 2014) hippocampal fields, rhythmic feedback inhibition from hippocampus long-range interneurons to MSDB (Alonso and Köhler, 1982; Tóth et al., 1993), as well as intrinsic resonant properties of hippocampal, entorhinal and medial septal neurons mediated by various voltage-dependent channels (Ca^2+^, K^+^, Na^+^) which provide membrane resonance and subthreshold oscillations at theta frequency (Kamondi et al., 1998; Evstratova et al., 2011; Hansen et al., 2014).

The theta rhythm appears to act as an internal clock: hippocampal pyramidal cells fire maximally at the trough of theta cycles, when perisomatic inhibition by parvalbumin (PV) and cholecystokinin (CCK) basket cells, as well as axo-axonic cells, is minimal; and different classes of GABAergic neurons have different preferred firing phases (Freund and Buzsáki, 1996; Csicsvari et al., 1999; Klausberger and Somogyi, 2008; Müller and Remy, 2014). Local GABAergic inhibition may fine tune the spike timing of pyramidal cells relative to theta [e.g., Ego-Stengel and Wilson, 2007; Geisler et al., 2007; Maurer and McNaughton, 2007; Royer et al., 2012; Grienberger et al., 2017; see the network model by Ferguson et al. (2015)]. Given the strong and reliable modulation of hippocampal activity by theta oscillations, behavior-related changes in the theta profile (e.g., spectral components) may play a crucial role in hippocampal dynamics.

Many structures activated during locomotion modulate theta (Vertes et al., 2004; Kiehn, 2016). Consistently, theta is primarily influenced by ambulatory signals, compared to e.g., optic flow or vestibular self-motion cues (Czurkó et al., 1999; Terrazas et al., 2005). Hence, numerous subcortical structures, which may not be directly involved in the actual generation of theta, have nonetheless been reported to modulate theta. These are part of the “synchronizing ascending system” (Vertes and Kocsis, 1997; Vertes et al., 2004; Orzeł-Gryglewska et al., 2015; Kiehn, 2016). Efferences of several subnuclei of the brainstem reticular formation and the mesencephalic locomotor region can trigger the emergence (onset, offset) and the regulation (power, frequency) of theta (Grastyán et al., 1965; Peck and Vanderwolf, 1991; Sinnamon, 1993; Vertes and Kocsis, 1997; Sńska and Kasicki, 1998; Jackson et al., 2008; Bland et al., 2016; Ma et al., 2017).

How is theta altered by locomotion? In the spectral domain, a 16 Hz oscillation, described as the second harmonic of theta (Harper, 1971), has been reported to emerge with increasing locomotion velocity (Figure 1C; Czurkó et al., 1999; Terrazas et al., 2005; Sheremet et al., 2016). More generally, a chain of harmonics significantly phase coupled to the theta oscillation appear to be related to the velocity of the animal (Sheremet et al., 2016). In the time domain, these speed-related nonlinearities are manifested as an increase in skewness and asymmetry in the shape of theta cycles (Buzsáki et al., 1983; Terrazas et al., 2005; Sheremet et al., 2016), which decrease along the septo-temporal axis of the hippocampus (Maurer et al., 2005; Hinman et al., 2011, 2013; Sheremet et al., 2016). In addition, the frequency of theta and its harmonics increase with running velocity (McFarland et al., 1975; Sńska and Kasicki, 1998; Maurer et al., 2005; Bender et al., 2015; Sheremet et al., 2016), in a consistent manner across the septo-temporal axis (Maurer et al., 2005; Sheremet et al., 2016). Intriguingly, theta phase shifts progressively from the septal to the temporal pole of the hippocampus, and thus theta can be seen as a “traveling wave” (Lubenov and Siapas, 2009; Patel et al., 2012).

Changes in theta power and frequency coincide with systematic changes in the spiking dynamics of pyramidal cells (Song et al., 2005b; Terrazas et al., 2005; Lu and Bilkey, 2010), and a strong correlation has been reported between theta nonlinearity and the intensity and timing of the spiking activity of both pyramidal cells and interneurons (Maurer et al., 2005; Sheremet et al., 2016). Thus, speed-related changes in theta oscillations have a strong impact on the precise spike timing of hippocampal cells. As described below, the precise phase at which spikes are emitted is directly related to the position of the animal in the environment (O'Keefe and Recce, 1993), which may constitute one of the clearest examples of temporal coding in the brain.

### 2.2. Theta Phase Precession

**Figure 2:**
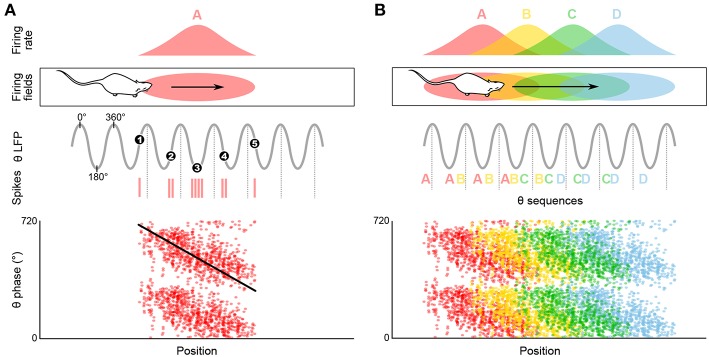
Temporal coding in the hippocampus and emergence of theta sequences. **(A)** Theta spike phase precession. Top, schematic representation of the temporal organization of successive place cell bursts relative to theta as a rat runs along a linear track. When the rat enters the firing field (pink ellipse), the cell fires near the end of the theta cycle, i.e., with a phase of ~360°. As the rat progresses through the field, bursts occur on earlier and earlier phases (red vertical ticks, action potentials; black numbered dots, mean burst times within individual theta cycles). Bottom, phase precession plot. For each spike, phase is represented as a function of position (tilted black line, best linear-circular regression line). **(B)** Top, four schematic overlapping firing fields. Middle, in their overlap region the cells discharge in sequence (A, AB, ABC, etc.) within each theta cycle, reflecting the order of field traversal at a compressed time-scale. Bottom, these past-present-future “sweeps” may require coordination between individual phase precessing cells.

As a rat walks through the firing field of a given place cell, the firing rate of this cell progressively increases until the animal reaches the center of the place field, then decreases until the animal leaves the field. Thus, the firing rate of the place cell appears to code for the location of the animal in the environment. Strikingly, O'Keefe and Recce (1993) discovered that spatial information can also be provided by the precise time at which the place cell fires relative to the ongoing theta rhythm. Indeed, the place cell fires at progressively earlier phases across successive theta cycles: firing occurs at late phases when the animal enters the place field, then progressively shifts back in phase as the animal progresses through the field, and finally occurs at the beginning of the theta cycle when the animal leaves the field. This phenomenon is referred to as “phase precession” (Figure 2A; O'Keefe and Recce, 1993). While the range over which spike theta phase precesses may appear to span as much as 360° when pooling data over multiple field crossings, single-trial phase range is typically ~180°, and never exceeds a full cycle (O'Keefe and Recce, 1993; Maurer et al., 2006; Schmidt et al., 2009). At a given (fixed) running speed, the rate of phase precession is inversely related to the size of the place field: in particular, place cells with small fields in the dorsal hippocampus phase precess faster than place cells with large fields in the ventral hippocampus (Maurer et al., 2005). On the other hand, phase precession slope increases with running speed, ensuring that the phase-position relationship is maintained regardless of running speed (Bose and Recce, 2001; Geisler et al., 2007).

Phase precession has been reported not only in all subfields of the hippocampus (Skaggs et al., 1996; Johnson et al., 2007; Mizuseki et al., 2009), but also in neighboring parahippocampal structures projecting to the hippocampus, including the subiculum (Kim et al., 2012) and medial entorhinal cortex (MEC, Hafting et al., 2008), as well as in downstream structures, such as the medial prefrontal cortex (Jones and Wilson, 2005) and ventral striatum (van der Meer and Redish, 2011). Understanding the mechanisms of hippocampal phase precession is thus expected to provide invaluable insights about a general coding scheme present in multiple brain areas (Harris, 2005; Malhotra et al., 2012). Recent evidence of phase precession in the human hippocampus (Qasim and Jacobs, 2016) further confirms that theta phase precession remains relevant beyond rodent species.

#### 2.2.1. Mechanisms of Phase Precession: Computational Models and Experimental Data

The neurophysiological mechanisms of phase precession are difficult to explore experimentally, but have been extensively investigated through computational modeling (for reviews, see Maurer and McNaughton, 2007; Burgess and O'Keefe, 2011). While no model appears to fully account for all aspects of phase precession, a continued dialogue between experimental and modeling approaches has provided constraints on potential generation mechanisms. Below, the three main classes of models of phase precession are briefly reviewed and discussed in light of experimental data.

**Figure 3:**
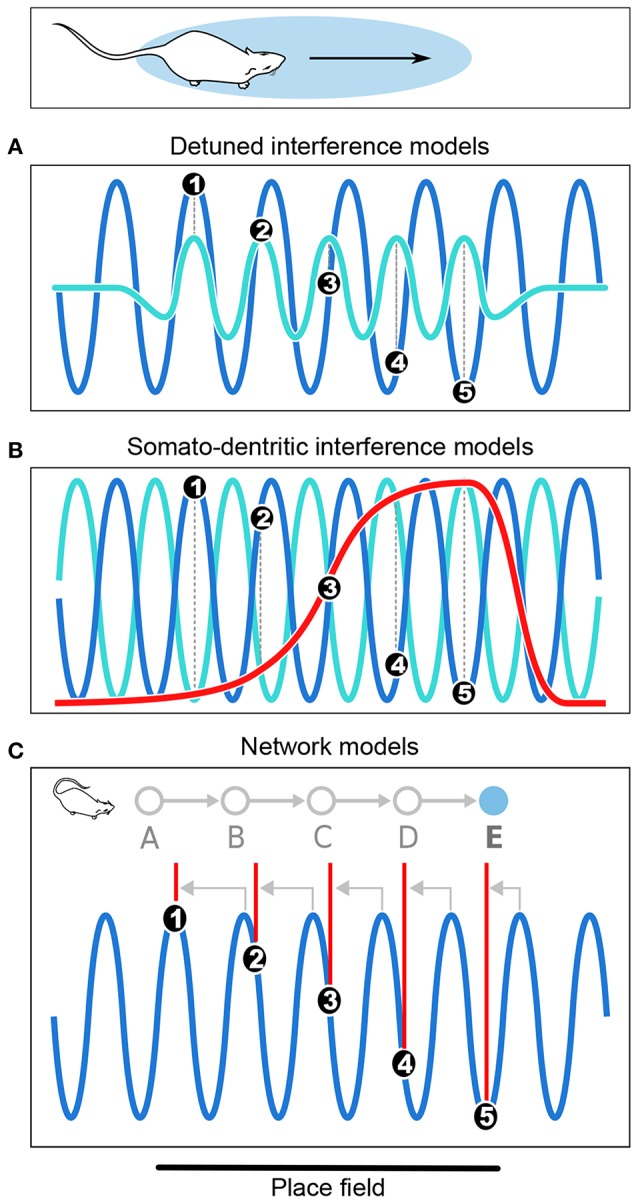
Computational models of phase precession. **(A)** In the detuned oscillators models, the membrane potential (*V*_*m*_) of the neuron (light blue) oscillates faster than the local theta rhythm (dark blue) within the firing field. Spike bursts (black dots numbered from 1 to 5) occur at positive peaks of *V*_*m*_ and thus anticipate theta cycles, resulting in phase precession. **(B)** In the somato-dendritic interference models with asymmetric excitation ramps, spikes are emitted whenever excitation (red) exceeds somatic inhibition (light blue), which is paced by theta oscillations (dark blue). Because excitation increases, it exceeds inhibition earlier and earlier, resulting in phase precession. **(C)** In the asymmetric connections model, successive place cells (labeled from A to E) with overlapping fields are connected (gray arrows) in such a way that activity propagates unidirectionally within the network. Thus, as the rat enters the field of cell E, it happens to be located at the center of the field of cell A. Cell A is therefore maximally excited, and transmits its activity via the chain of intermediate cells until it reaches cell E, which fires late in the theta cycle as a result of transmission delays. But as the rat progresses through the field, there are fewer and fewer intermediate steps and the transmission delays decrease, so that cell E fires earlier and earlier in the theta cycle. Note that in the present Figure, phase precession is shown from 360° to 180° to simplify the schematic representation.

The *detuned oscillators model* was originally proposed by O'Keefe and Recce (1993) in their initial report of phase precession, and remains one of the most elegant hypotheses to date^1^ (Figure 3A). This model considers a place cell that oscillates at a slightly faster frequency than theta. These two oscillators, synchronized at the beginning of the place field, slowly shift due to their frequency difference. Because the place cell fires at the peaks of its own membrane potential oscillation, i.e., its more depolarized states, this occurs earlier and earlier relative to the slower theta oscillation, which results in phase precession. In addition, the frequency of the membrane potential oscillation is assumed to be proportional to running speed, so that the phase advance of spikes reflects the distance traveled through the place field, rather than the time spent in the field. However, because the field size is maintained despite changes in running speed, it has been proposed that the frequency of both oscillators increased with running speed to compensate for the decrease in numbers of theta cycles at high running speed (Geisler et al., 2007, 2010). The model requires neurons with cosine directional tuning, which have been identified in the hippocampus, medial septum and anterior thalamus (Welday et al., 2011). Similar cells were documented in entorhinal cortex (Krupic et al., 2012), although the finding remains controversial (Krupic et al., 2015; Navratilova et al., 2016).

The generation sites of these two oscillators has remained unresolved. Intrahippocampal oscillators under the influence of medial septum and CA3 recurrent inputs are inconsistent with the finding that theta reset by brief single-pulse electrical stimulation of the ventral hippocampal commissure does not reinitialize phase precession and leaves the phase-position relationship intact (Zugaro et al., 2005). Extrahippocampal oscillators (e.g., Yamaguchi, 2003) would be supported by phase precession in MEC grid cells (in layer II and to a lesser extent in layer III) and conjunctive cells (in layer III) (Hafting et al., 2008; Reifenstein et al., 2012, 2014, 2016; Climer et al., 2013; Ebbesen et al., 2016), which persists during pharmacological inactivation of the hippocampus (Hafting et al., 2008). Conversely, hippocampal phase precession is disrupted after bilateral lesions of the MEC (Schlesiger et al., 2015, but see Ormond and McNaughton, 2015). Further, a recent study by Robinson et al. (2017) has shown that phase precession in object-responsive CA1 cells persists during transient optogenetic inactivation of the MEC, suggesting a possible complementary role for the LEC, which predominantly provides non-spatial information (Deshmukh and Knierim, 2011; Tsao et al., 2013). Several models have suggested that entorhinal oscillators might generate phase precession (Burgess et al., 2007; Climer et al., 2013; Jeewajee et al., 2014). On the other hand, relatively large phase shifts between EC and CA1/CA3 spikes (Mizuseki et al., 2009) remain at odds with the notion that phase precession could be directly transferred from the EC to the hippocampus. In addition, the mechanism proposed to account for phase precession in EC requires that entorhinal head direction cells signal movement direction, which is inconsistent with experimental findings (Cei et al., 2014; Raudies et al., 2015).

*Somato-dendritic interference models* (Figure 3B; Kamondi et al., 1998; Magee, 2001; Harris et al., 2002; Mehta et al., 2002; Losonczy et al., 2010) posit that the timing of action potentials is determined by the combination of oscillatory somatic inhibition and transient ramp-like dendritic excitation. As the animal enters the place field, the cell fires when excitation overcomes inhibition, which occurs earlier and earlier because of the ramp-like shape of the dendritic depolarization. The excitation ramp has been proposed to be either symmetrical (Harris et al., 2002) or asymmetrical (Mehta et al., 2002). The asymmetrical depolarization shape mirrors the asymmetrical development of place field with experience (Mehta et al., 1997, 2000). To remain consistent with the observation that spike phase decreases but does not subsequently increase, the symmetric ramp model posits that the place cell stops firing at the peak of the ramp. This was supported by *in vitro* data obtained following symmetrical-shaped depolarizing current injection through the pyramidal cell dendrites (Harris et al., 2002), which suggested that an adaptive mechanism allowed the cell to precisely stop firing at the ramp peak.

Both intracellular *in vitro* (Magee, 2001; Losonczy et al., 2010) and extracellular *in vivo* (Kamondi et al., 1998; Harris et al., 2002; Mehta et al., 2002) data are consistent with the somato-dentritic interference models. Notably, recordings of intracellular activity in awake behaving animals confirmed that within firing fields, the membrane potential of place cells had an asymmetric depolarization curve (Harvey et al., 2009; Epsztein et al., 2011; this was also observed in grid cells, Domnisoru et al., 2013; Schmidt-Hieber and Häusser, 2013). Epsztein et al. (2011) further documented an increase in the excitability of place cells during field traversal, compared to silent cells. While this supports the somato-dentritic interference models, the same studies also reported that spikes remain locked to the peaks of the membrane potential, a critical prediction of the detuned oscillators models. And because recent work has shown that even network connectivity models (see below) can account for these findings (Romani and Tsodyks, 2015), it becomes unclear whether intracellular data do discriminate between different classes of models of phase precession.

In the *network connectivity models*, contrary to the above cellular models in which no particular synaptic connectivity is assumed, phase precession results from transmission delays between asymmetrically connected cells (Figure 3C; Jensen and Lisman, 1996; Skaggs et al., 1996; Tsodyks et al., 1996; Romani and Tsodyks, 2015). According to these models, place cells are unidirectional and asymmetrically connected to each other. Feed-forward sensory inputs arriving when the animal reaches the end of the place field drive the cells to fire at early phases of the theta oscillation. Activity then spreads to place cells with fields located further along the trajectory (via asymmetric connections), which fire later in the cycle due to transmission delays. Hence, place cells fire earlier and earlier as the animal progresses through their field, resulting in phase precession. Asymmetric connections can be hard-wired (Dragoi and Tonegawa, 2011, 2013) or result from experience (Silva et al., 2015), consistent with the experience-dependent asymmetric expansion of place fields (Mehta et al., 1997, 2000). An alternative version of the model posits that phase precession results from phase differences between CA3 and entorhinal inputs (Chance, 2012; Fernández-Ruiz et al., 2017). In both cases, the proposed mechanisms are difficult to reconcile with the experimental finding that the relation between phase and allocentric spatial position changes during backward movement (Cei et al., 2014; Maurer et al., 2014). Further, this change occurs abruptly, ruling out the possibility that asymmetry could reverse via slow plasticity processes (Cei et al., 2014). Hence, these models may need refining to account for these experimental findings (but see Chadwick et al., 2015, 2016).

While phase precession has been mainly described in principal cells, there have been reports of phase precession in hippocampal interneurons, in particular those receiving putative monosynaptic connections from place cells recorded on the same tetrode. Within the field of their partner place cells, these interneurons share the same rate of phase precession (Maurer et al., 2006; Ego-Stengel and Wilson, 2007; Geisler et al., 2007). Because pyramidal cells generally fire before interneurons, the spatial selectivity of interneurons was suggested to be inherited from local pyramidal cells (but see Chadwick et al., 2016). Functionally, these interneurons could inhibit competing cell assemblies and therefore participate in the segregation of cell assemblies in space and time (Maurer et al., 2006; Cutsuridis and Hasselmo, 2012; Royer et al., 2012; Dupret et al., 2013). This is supported by recent data by Grienberger et al. (2017), who coupled LFP and intracellular recordings in head-restrained mice running on an enriched treadmill, and showed that optogenetic hyperpolarization of CA1 interneurons reduces both the rate and temporal coding of spatial location in CA1 place cells by suppressing out-of-field excitatory inputs.

#### 2.2.2. Functional Role of Phase Precession

While phase precession may code for allocentric position on linear tracks (O'Keefe and Recce, 1993; Jensen and Lisman, 2000), it is unclear how this code would generalize to two dimensional environments. Contrary to linear tracks, in open fields animals can enter place fields from multiple directions, and place cells precess in a similar manner regardless of the direction of motion. As a result, place cells fire at the same theta phase for multiple locations (Skaggs et al., 1996; Huxter et al., 2008). Thus, phase may instead code for the relative distance from the place field center (Jeewajee et al., 2014). Similarly, the reversal of the phase-position relation observed during backward travel (Cei et al., 2014; Maurer et al., 2014) indicates that phase does not code for the allocentric position of the animal, but more likely for the distance traveled through the field.

Phase precession has been observed in a variety of contexts including running on a wheel (Hirase et al., 1999; Pastalkova et al., 2008) or a treadmill (Royer et al., 2012; Cei et al., 2014; Grienberger et al., 2017), jumping (Lenck-Santini and Holmes, 2008), in virtual reality (Chen et al., 2013; Ravassard et al., 2013; Aghajan et al., 2015), and during fixation (Takahashi et al., 2014). While all of these conditions correspond to theta states, the animal does not necessarily change location. Consistently, several studies have reported object- and sound-modulated CA1 phase-precessing cells (Aronov et al., 2017; Robinson et al., 2017) and phase-precessing cells toward reward locations in the ventral striatum (van der Meer and Redish, 2011). This suggests that phase precession may not be uniquely linked to space, but rather to the full episodic component of the ongoing experience (e.g., reviewed in Jaramillo and Kempter, 2017). An even more drastic departure from the spatial coding hypothesis is the view that perhaps the main role of theta phase precession is the formation of neuronal activity sequences, segregated by theta cycles (Figure 2B; e.g., Skaggs et al., 1996; Schmidt et al., 2009).

### 2.3. Mechanisms of Theta Sequences

Whether phase precession is a cause or a consequence of the formation of theta sequences remains unresolved. As discussed above, phase precession could result from a variety of mechanisms that do not require sequential connectivity between neighboring place cells (*detuned oscillators* and *somato-dendritic interference* models). Independent initiation of phase precession in successive cells would automatically yield theta sequences (Skaggs et al., 1996). Thus, phase precession would be a causal mechanism of theta sequences. An alternative view is that hippocampal sequences result from specific patterns of connectivity within the hippocampal network (*network connectivity* models; see also Dragoi and Tonegawa, 2011). At the single cell level, this would be manifested as phase precessing spikes. Thus, phase precession would be a consequence of the formation of theta sequences.

Several studies have challenged the idea that phase precession could entirely account for theta sequences, by showing that a moderate addition of time jitter to experimental spike trains resulted in preservation of phase precession but a reduced prevalence of theta sequences (Dragoi and Buzsáki, 2006; Foster and Wilson, 2007; Itskov et al., 2008). This suggested that theta sequences required precise coordination between cell assemblies in addition to independent phase precession mechanisms. Two recent studies have provided further support for this view. Feng et al. (2015) analyzed single-trial phase precession and theta sequence formation in rats exploring a novel linear track, and reported that while single place cells exhibit phase precession from the first lap, theta sequences do not emerge until the second lap. Middleton and McHugh (2016) analyzed the temporal coding of CA1 and CA3 neurons during spatial navigation in a transgenic mouse model lacking CA3 synaptic transmission (CA3 tetanus toxin mouse line). They showed that the absence of CA3 inputs abolished the formation of theta sequences at the CA1 ensemble level despite the persistence of phase precession in individual neurons. They suggested that CA3 may be instrumental for temporal coordination of the CA1 neuronal population. In summary, the above studies point to an intermediate mechanism, where phase precession plays an important but non-exclusive role in generating theta sequences, complemented by temporal coordination processes which may require specific functional connectivity.

To formally test the requirement for additional coordination mechanisms, Chadwick et al. (2015) developed phenomenological models of independent and coordinated place cell activity during navigation, and compared their predictions in light of experimental data. They concluded that the independent coding model is sufficient to replicate the data previously interpreted as evidence for the coordinated assembly hypothesis. First, although it has been shown that predictions of individual CA1 place cell spike timing is improved by information conveyed by their peer cells (Harris et al., 2003), suggesting that these cells directly interact with each other consistent with the coordinated assembly hypothesis (Harris, 2005), Chadwick et al. (2015) showed that this no longer holds when both the modulation of firing phase by travel direction (Huxter et al., 2008; Climer et al., 2013; Cei et al., 2014; Jeewajee et al., 2014; Maurer et al., 2014) and the modulation of firing rate by running speed (e.g., Czurkó et al., 1999; Huxter et al., 2003; Geisler et al., 2007) are taken into account to predict the activity of a given cell. As a matter of fact, peer information even decreases the average prediction, consistent with the independent coding model. Second, the independent coding model also replicated the finding that spike jitter can degrade theta sequences but spare the phase-position relation (Dragoi and Buzsáki, 2006; Foster and Wilson, 2007; Itskov et al., 2008), and the covariance in firing rate between cell pairs across successive running laps (Foster and Wilson, 2007). Finally, the independent coding model also accounted for global remapping data that the coordinated assembly model failed to replicate.

Incidentally, both the independent coding and coordinated assembly models can account for internal sequence generation (Pastalkova et al., 2008; Wang et al., 2015), proposed to support cognitive processes. One potential issue with the independent coding model, which is not directly related to the generation of theta sequences, is that it remains unclear how sequences could reactivate during non-theta states (see also Chadwick et al., 2016). A possible solution would be that once theta sequences emerge due to independent phase precession, the repeated activation of successive neurons induces synaptic changes that result in asymmetric network connectivity; this would allow trajectory replay during sleep (Drieu et al., 2018; see section 3.3).

While direct interactions between pyramidal neurons may not be required, specific classes of hippocampal interneurons may be involved in coordinating sequential information. As stated earlier, despite the fact that interneuron activity usually displays weak spatial modulation (McNaughton et al., 1983), many interneurons fire spikes that precess in phase against the theta rhythm, tightly coupled to monosynaptically connected pyramidal cells (Maurer et al., 2006; Ego-Stengel and Wilson, 2007; Geisler et al., 2007). While this suggested that interneuron phase precession may be inherited from place cells (Maurer et al., 2006; Geisler et al., 2007), Chadwick et al. (2016) recently proposed an alternative model of theta sequences where phase precessing interneurons play a crucial role in coordination of place cell spike timing and the emergence of theta sequences. In their model, phase precession occurs dynamically whenever a place cell is driven by external inputs due to the transient functional coupling between place cells and interneurons. Consequently, phase precession and theta sequences are generated *de novo* within the network, and slow input sequences are automatically compressed into theta sequences in networks of interacting pyramidal cells and interneurons. This is consistent with the findings of Royer et al. (2012), who reported that optogenetic silencing of PV-expressing interneurons shifted the phase of pyramidal cell spikes toward the trough of theta (conversely, silencing of SOM-expressing interneurons increased the burstiness of pyramidal cell spikes without altering their theta phase). This suggests that somatic inhibition may play a major role in theta phase precession and segregation of cell assemblies (Royer et al., 2012; Dupret et al., 2013; Chadwick et al., 2016).

## 3. Theta Sequences, Navigation, and Memory

Both theoretical considerations and experimental data indicate that theta phase precession and theta sequences could underlie cognitive functions. While the seminal report of O'Keefe and Recce (1993) emphasized the probable role of phase precession in spatial coding, the authors also immediately recognized that phase precession placed conditions on the possibility that synaptic connections could be modified. This was shortly followed by suggestions that a critical function of phase precession was to bring neurons to fire within brief time windows conducive to synaptic plasticity (Burgess et al., 1994; Lisman and Idiart, 1995; Skaggs et al., 1996). Effectively, theta sequences would allow the hippocampal network to store temporally ordered information, such as events in their spatio-temporal context, thus constituting a substrate for episodic-like memory trace formation. Consistently, there is evidence that degradation of theta sequences impairs sleep replay underlying memory consolidation (Drieu et al., 2018), and is accompanied by memory deficits (Lenck-Santini et al., 2008; Robbe and Buzsáki, 2009). In addition, recent data indicate that theta sequences reflect ongoing cognitive processes (Wikenheiser and Redish, 2015b), suggesting that they could also be involved in planning and decision making.

### 3.1. Theta-Gamma Neural Code

**Figure 4:**
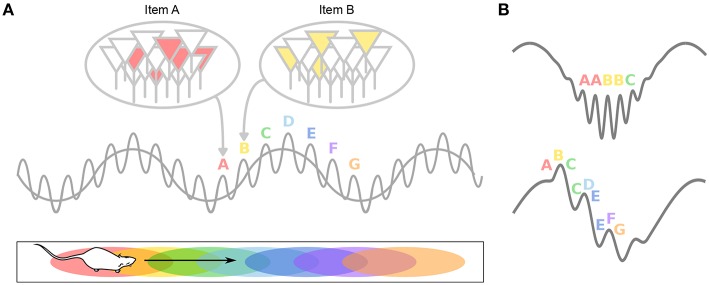
Theta-gamma neural code. **(A)** Individual memory representations (cell assemblies, identified by letters) are repeatedly activated within each new theta cycle. The gamma rhythm separates the respective representations in time. The number of gamma cycles per theta cycle determines the span of working memory. **(B)** If discrete locations (i.e., A–G) are encoded within individual gamma cycles, longer paths would be encoded during fast gamma than during slow gamma. Alternatively, slow (but not fast) gamma cycles encode sequences of locations, rather than single locations. This results in longer paths encoded during slow gamma than during fast gamma.

During theta states, nested gamma oscillations shape the activity of cell assemblies so that largely non-overlapping ensembles are sequentially activated in the 10–30 ms time windows of successive gamma cycles which occur at successive theta phases (Lisman and Idiart, 1995; Harris et al., 2003; Harris, 2005). This would enable the hippocampal network to represent four to eight items per theta cycle (corresponding to the number of nested gamma cycles), which has been related (Lisman and Idiart, 1995) to the “magic number” of 7 ± 2 items that humans can typically hold in short term memory (Miller, 1956). Hence, theta-gamma coupling has been hypothesized to form a neural code representing multiple items in a temporal frame (Figure 4A; Jensen and Lisman, 2005; Lisman and Buzsáki, 2008; Lisman and Jensen, 2013). Buzsáki (2010) even proposes the concept of a neural syntax, in which cell assemblies, the fundamental computational unit (letters), are arranged in specific orders by oscillations, external or internal excitatory inputs and local inhibition, in order to form words.

Evidence for a role of theta in information chunking has been provided in a number of experimental studies. By quickly changing light cues on the walls and floor of a recording chamber, Jezek et al. (2011) induced rapid switches between two perceived environments, and reported that CA3 representations flickered between the two maps. These network flickers tended to occur at the timescale of one or a few theta cycles. Notably, activity within a single theta cycle correlated with either environment, but rarely with both simultaneously, suggesting that theta cycles constituted temporal units of information. Similarly, Gupta et al. (2012) recorded CA1 activity while rats performed a decision task on a eight-shape maze with two reward sites, and reported that neural sequences expressed during single theta cycles reflected cognitive segments of the task. This supported the view that theta cycles may underlie the cognitive chunking of experience. Further, they reported that the length of the path encoded in a given theta cycle was directly related to the length of the cycle and to the number of gamma cycles it contained, suggesting that gamma cycles constitute information processing steps (see also Zheng et al., 2016).

The role of gamma oscillations in organizing spike timing has been supported by several recent studies. During theta oscillations, at least two types of nested gamma oscillations can occur in short bursts (Bragin et al., 1995), although it remains unclear whether these can occur within single theta cycles (Belluscio et al., 2012) or they are restricted to different theta cycles (Colgin et al., 2009). In CA1, slow gamma (gamma_S_, ~30–50 Hz) preferentially occurs on the descending phase of theta. It is thought to arise from CA3 and to propagate along the Schaffer collaterals to the *stratum radiatum*. Medium gamma oscillations (gamma_M_, ~50–90 Hz) tend to occur near the theta peak and may emerge from MEC inputs targeting the *stratum lacunosum-moleculare* (Bragin et al., 1995; Csicsvari et al., 2003; Colgin et al., 2009; Belluscio et al., 2012; Schomburg et al., 2014; Buzsáki and Schomburg, 2015).

Takahashi et al. (2014) studied hippocampal responses during fixation periods in a delayed spatial-alternation task. Neurons selective for the alternation sequence phase precessed during the entire fixation period, shifting their spikes from the peak to the trough of theta oscillations. Concomitantly, gamma_M_ power (60–90 Hz) arising at the theta peak decreased, while gamma_S_ power (30–45 Hz) arising at the theta trough increased toward the end of the fixation period. The authors hypothesized that CA1 received predominant inputs from the EC at fixation onset, providing information about ongoing context. This signal would then elicit auto-associative intra-hippocampal processing in CA3 to recall the required action, or to look up in memory the appropriate task sequence given the ongoing context. This scenario, reminiscent of the model of Chance (2012), is consistent with the finding that gamma_S_ power is reduced and theta sequences are disrupted in inducible KO mice lacking CA3 inputs (Middleton and McHugh, 2016). Different gamma frequency bands would thus reflect different types of information processing, e.g., externally cued vs. internally generated.

This is in line with the notion that theta cycles have two complementary functional modes (Hasselmo et al., 2002): an encoding phase at the peak of theta (as recorded from CA1 pyramidal layer), largely mediated by EC inputs, and a retrieval phase at the theta trough mediated by CA3 recurrent activity. Incidentally, this could explain why Jezek et al. (2011) reported a better separation of the two hippocampal maps during the second half of the theta cycle, when CA3 retrieval mode would dominate. A similar dissociation would be expected in the study of Gupta et al. (2012), where theta sequences representing trajectories ahead of the animal, reflecting memory retrieval, could be linked to higher gamma_S_ band power, whereas trajectories behind the animal, reflecting memory encoding, could be linked to higher gamma_M_ band power. Unfortunately, this was not investigated, but two recent studies reported a similar dissociation in rats running on a linear track.

In the first study, Bieri et al. (2014) found that late theta phases were dominated by gamma_S_, when spikes appear to anticipate the upcoming field location (“prospective” firing, Battaglia et al., 2004). In contrast, early phases were dominated by gamma_M_, when spikes lag behind field location (“retrospective” firing). These results suggest that alternating gamma_S_ and gamma_M_ states allow the hippocampus to switch between prospective and retrospective modes, possibly to prevent interference between memory retrieval and encoding.

In the second study, Zheng et al. (2016) performed Bayesian reconstruction of the trajectory of the animals at the theta timescale, and showed that theta sequences more accurately represented ongoing trajectories during theta cycles dominated by gamma_M_ than during theta cycles dominated by gamma_S_ (Figure 4B). Gamma_S_-associated theta sequences were found instead to represented relatively long paths extending ahead of the current location. In these sequences, spike phases relative to gamma_S_ also conveyed spatial information (Senior et al., 2008). This again argues that encoding and retrieval could be mediated by different gamma subtypes: relatively long gamma_S_ periods may allow successive items within the stored sequence to be retrieved in a time-compressed manner; in contrast, short periods of gamma_M_ would encode spatial memories in real time and may ensure chunking of cell assemblies. Note however that in this study, entire theta cycles were considered to contribute either gamma_S_ or gamma_M_ sequences (depending on the overall dominating gamma band), but not both (although see e.g., Belluscio et al., 2012), leaving unexplored the possibility that encoding and retrieval could both occur within single theta cycles (Hasselmo et al., 2002).

The spatial strategy of the animal may also bias the balance between gamma_S_ and gamma_M_. Cabral et al. (2014) trained mice to find food rewards in a five-arm maze. During training, mice always started exploring the maze from the same location. But during test trials, they started from a different location, which allowed to assess if the animals used a place strategy (navigation to the same place in space) vs. a sequence strategy (performing the same series of left or right turns). When the mice used a sequence strategy, gamma_S_ was most prominent and place maps were more similar to training trials (after realignment), compared to when the mice used a place strategy, when spikes emitted during gamma_S_ and gamma_M_ periods contributed equally to the hippocampal representation. A possible interpretation is that during sequence strategy trials, the hippocampal network retrieves sequences of cell assemblies stored in CA3, while during place strategy trials, hippocampal activity would be equally driven by the recall of memory stored in CA3 and by landmark-related sensory information conveyed by the EC.

### 3.2. Theta Sequences and Trajectory Planning

**Figure 5:**
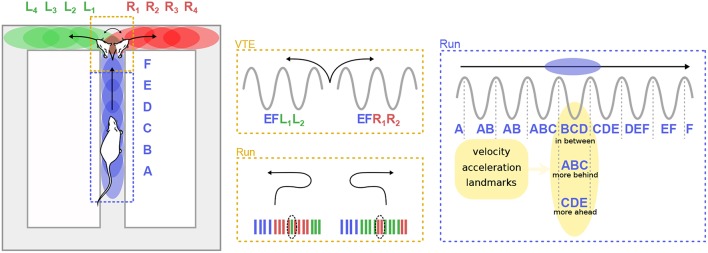
Theta sequences and trajectory planning. **(Left)** A rat runs on the central arm of a maze (blue dashed box) and must decide whether to turn left of right at a choice point (red dashed box). Letters represent hippocampal cell assemblies and colored ellipses illustrate their respective firing fields on the maze. Center. During deliberation (vicarious trial and error, VTE) at the choice point, sequences of cell assemblies alternatively sweep toward the two possible paths (top). Extra-field firing can occur before the animal turns back toward the choice point during error correction trials (bottom). **(Right)** When the animal runs along a segment of the maze, theta sequences can represent positions centered on the animal (“in-between”), behind the animal (“more behind”) or ahead (“more ahead”). The spatial representation embedded in theta sequences is influenced by multiple factors, including the velocity and acceleration of the animal, as well as the location of surrounding landmarks.

As hinted previously, theta sequences do not appear to be entirely driven by sensori-motor signals. For instance, Gupta et al. (2012) reported that “look-ahead” or “look-behind” theta sweeps were preferentially expressed in different maze segments, and were modulated by the running velocity of the animal (Figure 5). Bieri et al. (2014) found that prospective spikes tended to occur when rats were leaving reward locations (the ends of a linear track), whereas retrospective spikes tended to occur as rats approached reward locations.

Convincing and direct evidence that cognitive mechanisms can regulate theta sequences was provided by Johnson et al. (2007), who recorded CA3 activity in rats performing two different versions of the T-maze decision task. At decision points, when the rat had to choose to turn left or right, theta sequences quickly and repeatedly swept forward toward the reward sites, alternatively representing the two possible paths. Similar anticipatory sequences occurred upon recovery from an error (Figure 5). The animals performed two different tasks: a multiple-T task, in which reward locations were fixed within a given session but changed from day to day, and a cued-choice task, in which a tone indicated for each lap which arm would be rewarded. In the multiple-T task, the number of sweeps down the unrewarded arm decreased across trials. In contrast, in the cued-choice task, sweeps kept representing both directions throughout the session. Interestingly, at the decision point, the rats often expressed a typical behavior, consisting in pausing and orienting the head toward the left or right corridors, as if deliberating over the choice. This process is referred to as “vicarious trial and error” (VTE; Muenzinger and Gentry, 1931). Subsequent studies further linked VTE to decision-making processes (Schmidt et al., 2013; Amemiya and Redish, 2016; for a complete review see Redish, 2016). Of particular note, Wikenheiser and Redish (2015b) showed that theta sequences predicted longer trajectories when the animal was actually planning to reach a more distant goal. Thus, theta sequences also convey information about ongoing goals or intentions.

### 3.3. Theta Sequences and Memory

Because the mechanisms underlying theta sequences remain poorly understood and may involve many interacting factors (including single-cell properties, synaptic connectivity patterns, local inhibition, theta-paced inputs, etc.), interfering with theta sequences is challenging. To the best of our knowledge, only three studies have reported task performance deficits linked to a degradation of theta sequences.

In the first study, Lenck-Santini et al. (2008) investigated hippocampal theta dynamics in rats, following *status epilepticus*, a condition that leads to mesial temporal lobe epilepsy (MTLE). These rats had altered phase precession and temporal organization of firing among pairs of neurons, along with deficits in spatial learning in the Morris water maze. This suggests that memory impairments stemmed from impaired temporal coding, consistent with a role for theta sequences in normal memory function.

**Figure 6:**
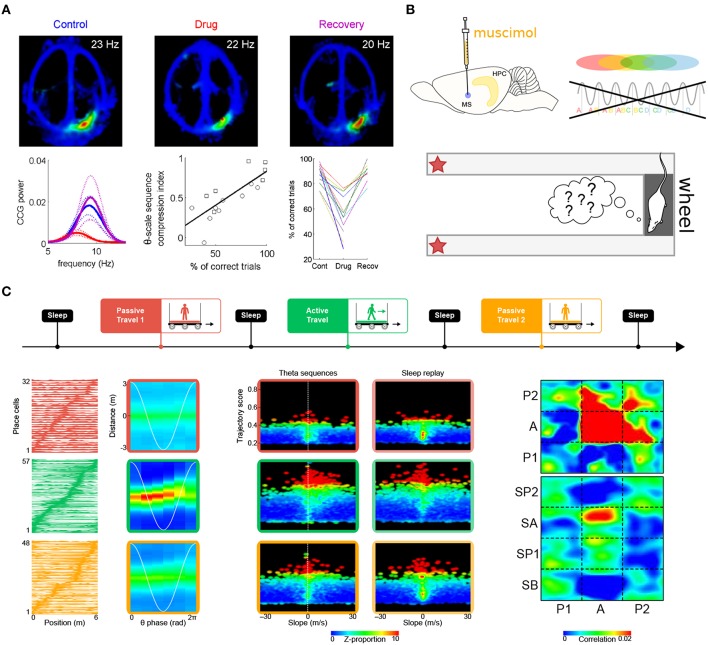
Theta sequences and memory. **(A)** Selective perturbation of phase precession, but not firing maps, by injection of cannabinoid receptor agonist CP55940 induces performance deficits in a spatial memory task. Top. Firing maps for an example place cell recorded in control (left), drug (center), and recovery (right) conditions. Bottom left. Average power of the cross-correlograms for all neuron pairs with overlapping fields during control (blue), drug (red), and recovery conditions (magenta; dotted lines: s.e.m.) Bottom center. Correlation between behavioral performance (percentage of correct trials) and theta-scale sequence compression index in one rat. The different symbols refer to data obtained in two different experiments. Bottom right. Average performance (correct trials) for all sessions and rats. **(B)** Medial septum inactivation by muscimol infusion impairs theta sequences and behavioral performance. **(C)** Perturbation of theta sequences impairs subsequent sleep replay. Top. Experimental protocol. The rats traveled on a model train. In successive sessions, a miniature treadmill was either turned off (passive travel; red and yellow) or on (active travel; green) to selectively disrupt or maintain theta sequences, while leaving behavioral timescale sequences intact. Bottom left. Firing fields (left column) remained stable in all conditions (red, green and yellow curves), but theta sequences (right column) emerged only during active travel (green box). Bottom center. Trajectories represented by hippocampal sequences were assessed using a combination of measures, namely trajectory score (linearity) and slope (velocity). During passive travel and subsequent sleep (red and yellow boxes), a very low proportion of both theta and replay sequences emerged in the hippocampal network (upper right quadrants are mostly black). On the contrary, during active travel and subsequent sleep (green boxes), a dramatically higher number of theta sequences and replay events were observed (red dots in upper right quadrants). Bottom right. Similarity of theta and replay sequences (P1, P2: passive travel sessions; A: active travel session; SA, SP1, SP2: sleep following respective travel sessions). Theta sequences were clear and self-consistent only during active travel (red zone at [A,A]). Sleep replay mirrored theta sequences only following active travel (red zone at [A,SA]). Panel **(A)** adapted from Robbe and Buzsáki (2009); panel **(C)** adapted from Drieu et al. (2018), with permission from AAAS.

In the second study, Robbe and Buzsáki (2009) examined the involvement of cannabinoid receptors in the dynamics of the hippocampal network (see also Robbe et al., 2006). Rats were trained to perform a hippocampus-dependent delayed spatial alternation task (Figure 6A). Administration of the cannabinoid receptor agonist CP55940 profoundly and reversibly impaired task performance. While the hippocampal place representation remained essentially intact, both theta phase precession and spike-timing coordination between place cells at the theta timescale were reversibly reduced. Notably, the percentage of correct trials on the task was correlated with the theta-scale pairwise coordination (assessed as the “compression index,” i.e., the ratio of the distance between the place fields of pairs of place cells and the average time lag between their spike trains at the theta timescale). These results strongly argue for a role of phase precession and theta sequences in task performance. One potential caveat however is that CP55940 administration decreases ripple power (Robbe et al., 2006). Since place cell activity during awake ripples may play a crucial role in working memory (Jadhav et al., 2012), it is conceivable that the performance deficit could also result from altered place cell activity during awake ripples.

Wang et al. (2015) also reported impaired task performance following perturbation of theta-timescale activity (Figure 6B). In rats performing a delayed spatial alternation task, medial septum inactivation by muscimol injection abolished theta sequences both on the maze and in the wheel where the rats ran during delay periods (although measures of theta cycles and therefore theta sequences may not be as reliable when theta amplitude vanishes following muscimol injection). On the other hand, the place-cell representation was spared when the rats were navigating the maze. Yet, performance was impaired. This suggests that theta sequences normally formed on the maze would have been necessary to achieve optimal levels of performance. One cannot entirely rule out that the observed performance deficits could be directly due to disruption of theta oscillations (Pan and McNaughton, 1997; Hasselmo et al., 2002) induced by medial septum inactivation (~80% decrease in theta power, Wang et al., 2015).

In summary, the above studies are consistent with the view that theta sequences are involved in the formation and recall of spatial memory, possibly by enabling the synaptic changes that underlie the storage of initial memory traces (Burgess et al., 1994; Skaggs et al., 1996). Yet, an alternative possibility is that these performance deficits resulted from impairments in planning and decision making, which also appear to involve theta sequences (Johnson et al., 2007; Schmidt et al., 2013; Wikenheiser and Redish, 2015b; Amemiya and Redish, 2016), rather than from deterioration of learning and memory processes. Thus, are theta sequences actually necessary for memory formation and recall? Bittner et al. (2017) recently uncovered a novel form of synaptic potentiation occurring at the behavioral timescale, and proposed that this could suffice to store entire sequences of events that took place several seconds before and after initiation of plateau-potentials in CA1 pyramidal cells. This effectively undermines the theoretical framework that theta sequences are required to bring spike trains in close temporal proximity and enable synaptic potentiation, underlying hippocampus-dependent memory encoding.

Because during exploration, theta sequences are nested within behavioral timescale sequences, disentangling their respective roles in memory encoding requires a protocol that instantly and selectively blocks or releases theta sequences throughout the hippocampal system, but spares the firing fields of place cells (and therefore, behavioral timescale sequences). Our previous work has established that passive travel alters phase precession in most place cells (see also Song et al., 2005a; Terrazas et al., 2005), except if the rats actively run on a treadmill while being transported (Cei et al., 2014). Importantly, switching the treadmill on or off does not induce global remapping of place cells. We have recently leveraged this protocol to selectively impair phase precession (treadmill off) or leave it intact (treadmill on), while maintaining the spatial layout of place fields in the environment (Drieu et al., 2018). The rats were tested in an entirely novel environment, to stimulate spatial learning, network coordination and synaptic plasticity (Li et al., 2003; Leutgeb et al., 2004; Cheng and Frank, 2008; O'Neill et al., 2008), as well as to ensure that previously formed memory traces would not bias our observations. As expected, impaired phase precession during passive travel (treadmill off) was accompanied by an almost complete absence of theta sequences. On the other hand, behavioral timescale sequences were unaffected. During subsequent sleep, the hippocampal network failed to replay trajectories. Only following “active” travel (treadmill on), when theta sequences emerged nested within behavioral timescale sequences, did the hippocampal network generate genuine replay events during sleep (Drieu et al., 2018) (Figure 6C). This indicates that theta sequences play a causal role in establishing initial memory traces, which can later be reinstated during sleep for memory consolidation.

Note that in this study, the functional role of theta sequences was not assessed from behavior (task performance), but from the occurrence of dynamic network patterns with a well-documented function, namely trajectory replay underlying memory consolidation. As a consequence, this alleviates the ambiguity discussed earlier, that behavioral impairments could result either from memory deficits, or from impairments in planning and decision making.

Interestingly, additional evidence for a link between theta sequences and sleep replay was recently provided by Muessig et al. (2019), who recorded from rat pups at different post-natal stages. They reported that before weaning (P21), ripple-associated activity represents stationary locations in space, and does not start to represent *bona fide* trajectories until theta sequences progressively emerge between P17 and P32.

Finally, over the last 15 years, an ever growing number of studies have addressed the network substrates of memory using causal approaches. However, these studies have generally failed to consider the temporal dimension of neuronal activity, such as manifested in cell assemblies and sequences. Experimental exploration of engrams (Semon, 1921) has benefited from the development of optogenetics (Boyden et al., 2005) and gene expression control techniques combined with immediate early gene (IEG) expression signals, to artificially manipulate memory traces (e.g., Reijmers et al., 2007; Liu et al., 2012; Cowansage et al., 2014; Redondo et al., 2014; Trouche et al., 2016). These strategies allow tagging and subsequent manipulation of neuronal populations activated during learning (for recent reviews, see Silva et al., 2009; Tonegawa et al., 2015; Guan et al., 2016; Kim et al., 2016; Poo et al., 2016). Thus, selective activation or inhibition of tagged neuronal ensembles have been shown to induce (Liu et al., 2012; Cowansage et al., 2014; Kim et al., 2014; Redondo et al., 2014) or block (Han et al., 2009; Trouche et al., 2016) the expression of memory, respectively.

One important issue with most of these studies is that they artificially activate neurons in a hyper-synchronous manner, and neglect any fine-timescale temporal information embedded in endogenous spiking patterns. In addition, because the time window for genetic labeling of neuronal networks ranges from several hours to days, the tagged population is likely to include both memory-specific neurons as well as numerous irrelevant neurons that were activated by unrelated events during the relatively long labeling time window. In any case, it remains unclear whether the tagged neurons represent a memory trace or constitute relays toward the relevant ensembles. For example, memory recall could actually result from downstream circuit computations rather than from the experimental activation of the tagged neurons *per se*. This would potentially restore the temporal patterns obliterated by the synchronous experimental stimulation. A parallel can be made with fRMI studies showing robust and consistent activation patterns in response to similar stimuli, despite the low temporal and spatial resolution of the technique (Guest and Love, 2017). Thus, while certain types of computation can still be detected or elicited even without having access to the fine time scale of neuronal computation, it is uncertain that they reflect actual brain computation during learning and memory. Further experimental and computational work will undoubtedly shed light on the complex issue of the temporality of circuit computations underlying memory.

## 4. Hippocampal Sequences During Exploration: Distinct and Complementary Functions

### 4.1. Awake Replay During Sharp-Wave Ripples

**Figure 7:**
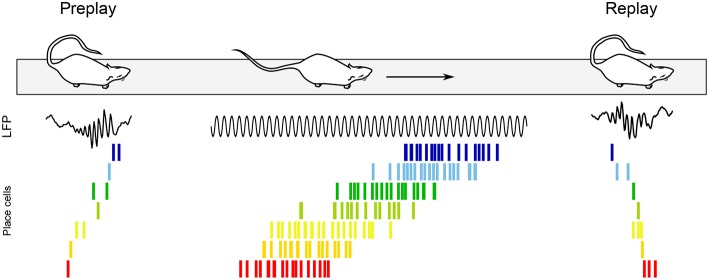
Awake replay. Place cell sequences experienced during behavior are generated in both the forward and reverse direction during awake SPW-Rs (Diba and Buzsáki, 2007). Spike trains of place cells on the track are shown before, during and after a single traversal. Sequences that occur during track running are reactivated during SPW-Rs both before and after the run, when the rat remains immobile. Forward preplay **(Left)** tends to occur as the animal is getting ready to run on the track, while reverse replay **(Right)** tends to occur shortly after run. Black traces, CA1 local field potential.

Theta sequences are not the sole pattern of sequential activity in the awake hippocampus. While offline replay of neuronal sequences was first documented during sharp-wave ripples (SPW-Rs) of slow wave sleep (Pavlides and Winson, 1989; Wilson and McNaughton, 1994; Lee and Wilson, 2002), replay also occurs during SPW-Rs of quiet awake states, such as when the animal pauses in an environment (Figure 7; Buzsáki, 1989; Foster and Wilson, 2006; Jackson et al., 2006; O'Neill et al., 2006; Csicsvari et al., 2007; Diba and Buzsáki, 2007; Dupret et al., 2010). Foster and Wilson (2006) recorded CA1 pyramidal cell activity while rats ran back and forth between reward sites, and showed that compared to the location of their firing fields on the track, place cells tended to reactivate in reverse order during awake replay, contrary to replay during slow wave sleep (although more recent studies have since documented overlooked reverse replay during sleep as well; see e.g., Wikenheiser and Redish, 2013; Silva et al., 2015; Grosmark and Buzsáki, 2016; Ólafsdóttir et al., 2016; O'Neill et al., 2017; Drieu et al., 2018). Replay events were more readily observable on a new track, where they occurred even after the very first lap (Foster and Wilson, 2006). In addition, Diba and Buzsáki (2007) reported that replay tended to occur in the reverse order upon arrival at the reward site, but in the forward order (“preplay”) when rats then faced the track in anticipation of the next run (Figure 7). Interestingly, the authors documented a strong correlation between the temporal offsets of neuron pairs during forward events (preplay) and during theta oscillations when the animals were running between reward sites. In contrast, theta sequences and reverse replay events were less strongly correlated, suggesting a selective link between offline preplay and neural activity during run. The balance between replay and preplay may also depend on task contingencies, as Ólafsdóttir et al. (2017) found a greater proportion of forward replay immediately (< 5 s) before and after movement or during short stops (< 10 s), but no directional bias during longer periods of immobility (although one cannot exclude that the apparent inconsistency with the results of Diba and Buzsáki (2007) may stem from the limited number of replay events examined in the Ólafsdóttir et al. (2017) study).

Foster and Wilson (2006) hypothesized that reverse replay could be associated with a slowly decaying dopamine signal, effectively storing a value gradient path that the animal could follow during subsequent goal-finding behavior. Conversely, forward preplay has been hypothesized to be involved in goal-directed planning (Diba and Buzsáki, 2007). Accordingly, awake replay can be influenced by reward locations (Foster and Wilson, 2006; Singer and Frank, 2009; Ambrose et al., 2016), contributes to mapping novel environments (O'Neill et al., 2006; Cheng and Frank, 2008; Roux et al., 2017), helps establishing goal-related cell assemblies (Dupret et al., 2010; Csicsvari and Dupret, 2014), supports decision making (evaluation of choices, prospection and planning) (Pfeiffer and Foster, 2013; Singer et al., 2013; Yu and Frank, 2015), and plays a role in fear (Wu et al., 2017) and episodic-like (Takahashi, 2015) memory retrieval. In addition, awake replay can span multiple SPW-R events to represent extended trajectories (Davidson et al., 2009) and link familiar paths into novel trajectories never experienced before (Gupta et al., 2010). Finally, selective suppression of awake SPW-Rs during a spatial alternation task fails to impair memory consolidation, and instead appears to alter working memory and decision making (Jadhav et al., 2012). In summary, awake replay has been proposed to mediate both spatial and non-spatial functions (see O'Neill et al., 2010; Buhry et al., 2011; Carr et al., 2011; and for a recent review Roumis and Frank, 2015).

Because both forward and reverse replay have a tendency to start at the current location of the animal, awake replay has been proposed to be biased by a residual, place-selective spatial tuning drive (Foster and Wilson, 2006; Csicsvari et al., 2007; Diba and Buzsáki, 2007). However, awake replay can also be modulated by non-local cues such as rewards (Pfeiffer and Foster, 2013; Singer et al., 2013) and contextual memories (Takahashi, 2015; Wu et al., 2017), or even be initiated at remote locations (Davidson et al., 2009; Karlsson and Frank, 2009; Gupta et al., 2010). This is at odds with the seminal model which posits that replay (in particular, reverse replay) can be accounted for by decreasing excitability over time (Buzsáki, 1989; Foster and Wilson, 2006). Instead, it has been proposed that awake replay could emerge from auto-associative properties of the CA3 network, in which the activation of a single cell can initiate the reactivation of an entire trajectory sequence due to recurrent connectivity established or reinforced during theta-state exploration (Lee and Wilson, 2002; O'Neill et al., 2008; Dupret et al., 2010; Silva et al., 2015), similar to the classical model of sleep replay. In turn however, this model cannot account for the (weak) link between the time spent exploring different areas of the environment and the propensity of replay to represent the corresponding trajectories (Gupta et al., 2010; but see O'Neill et al., 2008), the construction of never-experienced sequences (Gupta et al., 2010), nor the differential balance of forward and backward replay across behavioral states (Wikenheiser and Redish, 2013). It is therefore likely that replay results from a combination of these mutually non-exclusive mechanisms. Hence, Atherton et al. (2015) have suggested that the lingering excitability model largely dictates local replaying sequences during awake behavior, while the synaptic plasticity model contributes to subsequent nonlocal awake replay (and sleep replay). Finally, neuromodulatory activity may also exert an important role (Atherton et al., 2015): dopamine release during novel maze exploration and reward-driven spatial tasks may promote the strengthening of new place cell assemblies and place-reward associations (Benchenane et al., 2010; Dupret et al., 2010; McNamara et al., 2014; de Lavilléon et al., 2015; Ambrose et al., 2016; Takahashi et al., 2016; Takeuchi et al., 2016), while cholinergic tone may influence the direction of replay in different behavioral states (Marrosu et al., 1995; Csicsvari et al., 2007; Wikenheiser and Redish, 2013; Saravanan et al., 2015).

### 4.2. Functional Dissociation Between Theta Sequences and Awake Replay?

The above discussion suggests a functional dissociation between theta sequences and awake replay, where theta sequences would store initial memory traces about ongoing experiences as well as recall previously formed memories and enhance them to include goal-directed information, while awake replay would play an instrumental role in learning goal locations and planning upcoming trajectories. Complementing these mechanisms, sleep replay would sustain memory consolidation. As it turns out, a closer examination of the available data suggests a more complex picture. Below, we point out some of the current inconsistencies and overlooked findings.

#### 4.2.1. Memory Maintenance and Retrieval

Wang et al. (2016) inactivated the medial septum in rats trained on a delayed spatial alternation task. They found that theta sequences were disrupted and performance was impaired. Surprisingly, awake replay was preserved. The authors concluded that theta sequences on the maze (and in the running wheel during delays), but not awake replay, are required to recall episodic memories even in well-trained animals. Alternatively, awake replay would be required only for initial learning of a task but not once the environment and the task have become familiar. This would be consistent with the finding that suppression of awake SPW-Rs during the first 8 days of learning impairs working memory in a spatial alternation task (Jadhav et al., 2012). Yet another possible explanation for the apparently predominant role of theta sequences over awake replay may be that the rats spent the delay period running in the wheel, which may have favored neuronal events occurring in the theta state. In any case, these results still argue that theta sequences and awake replay do not play equivalent, interchangeable roles.

Wu et al. (2017) trained animals in a foot-shock avoidance task and reported that avoidance of the shock zone was concomitant with awake replay, but not theta sequences, of paths leading from current position to the foot-shock zone. This further supports distinct functional roles for theta sequences and awake replay in long-term contextual memory retrieval.

#### 4.2.2. Planning and Decision-Making

Another notable difference between theta sequences and awake replay is that theta sequences seem to be restricted to short distances always encompassing current location, occasionally extending to further locations (Johnson et al., 2007; Wikenheiser and Redish, 2015b), whereas awake replay can span much longer distances (up to ≥10 m; Davidson et al., 2009) and be decoupled from current experience (Karlsson and Frank, 2009; Gupta et al., 2010). Therefore, while theta sequences may be relevant for spatial memory, in particular to allow trajectory encoding and retrieval (Hasselmo and Eichenbaum, 2005; Dragoi and Buzsáki, 2006; Foster and Wilson, 2007; Robbe and Buzsáki, 2009; Wang et al., 2015; Drieu et al., 2018), awake replay may be necessary to associate paths and events disconnected in time and space, allowing flexibility and adaptive behaviors (Gupta et al., 2010).

During deliberative behavior at a choice point (Muenzinger and Gentry, 1931; Redish, 2016), theta sequences reflect possible paths (Johnson et al., 2007). Papale et al. (2016) reported that the occurrence rate of VTE at the choice point was inversely correlated to that of SPW-Rs at the reward sites on the same lap. Further, the SPW-R rate on a given lap inversely correlated with the likelihood of subsequent VTE events. Finally, interruption of SPW-Rs during learning led to an increased prevalence of VTE. This suggests that SPW-Rs are involved in ongoing consolidation, while VTE sequences participate in a decision-making process. On the other hand, SPW-Rs are also known to occur at decision points during learning (Jadhav et al., 2012), which could reflect an implication in decision-making processes, blurring the proposed functional distinction between theta sequences and awake replay. Disentangling these matters is further hindered by the fact that ripple disruption in Jadhav et al. (2012) was not triggered with spatial specificity: ripples were disrupted irrespective of the location of the rat. Therefore, the specific functional contribution of ripples occurring at decision points remains to be elucidated.

#### 4.2.3. Learning and Consolidation

Both theta and SPW-Rs are propitious to synaptic potentiation between sequentially activated cell assemblies (Skaggs et al., 1996; Magee and Johnston, 1997; Markram et al., 1997; Dragoi et al., 2003; Wójtowicz and Mozrzymas, 2015; Sadowski et al., 2016; Cobar et al., 2017; Drieu et al., 2018). Therefore, in principle neuronal sequences in both states could lead to learning and reinforcement of sequential activity.

In the machine learning literature, reverse replay has been repeatedly reported to enhance reinforcement learning, in particular temporal difference learning (Cazé et al., 2018; Mattar and Daw, 2018). Interestingly, awake replay during brief pauses in exploration and at reward sites largely occur in reverse order (O'Neill et al., 2008; Wikenheiser and Redish, 2013), identifying a potential mechanisms to support learning of trajectories and goal locations (Singer et al., 2013). In addition, working memory deficits have been observed following suppression of SPW-Rs that occurred mainly at reward locations (Jadhav et al., 2012). Theta sequences on the other hand mainly reflect currently experienced trajectories in the forward order. Yet, a small fraction of theta cycles do contain significant reverse theta sequences (Gupta et al., 2012; Zheng et al., 2016; our own unpublished observations). The function of reverse theta sequences remains unclear. Similarly, although this has been overlooked in initial descriptions of sleep replay, recent studies have reported abundant reverse replay during sleep as well (Wikenheiser and Redish, 2013; Silva et al., 2015; Grosmark and Buzsáki, 2016; Ólafsdóttir et al., 2016; O'Neill et al., 2017; Drieu et al., 2018). Again, these considerations dim the usual theoretical distinction between (forward) theta sequences for memory encoding, (reverse) awake replay for goal learning, and (forward) sleep replay for memory consolidation.

An additional complexity is that SPW-R incidence and replay strength and accuracy vary according to the length, shape and complexity of the maze, as well as the cognitive demand of the task (Jackson et al., 2006; Dupret et al., 2010; Pfeiffer and Foster, 2013; Singer et al., 2013). Increases in both SPW-R occurrence rate and replay strength can even be observed in familiar environments (Jackson et al., 2006; Dupret et al., 2010; Pfeiffer and Foster, 2013; Singer et al., 2013), perhaps because awake replay helps stabilize hippocampal place fields when new configurations are learned within a familiar map (Roux et al., 2017).

## 5. Conclusion

Sequences of hippocampal cell assemblies formed during individual theta cycles represent the time-compressed ongoing trajectory of the animal. These theta sequences are thought to emerge from ensembles of phase precessing cells, although it is currently unclear whether this requires additional coordination between participating neurons. Numerous computational models have proposed cellular and network mechanisms to account for theta sequences, but the precise neurophysiological mechanisms of phase precession remain elusive. While initial reports emphasized the presence of sequential activity during single theta cycles, recent studies have increasingly recognized that a notable fraction of theta cycles contain non-canonical activity, including seemingly random noise and even reverse sequences.

Theta sequences chunk information into time windows conducive to classical synaptic plasticity mechanisms, and appear to support the initial storage of memory traces. Within familiar environments, theta sequences allow the maintenance (and possibly the update) of planned trajectories in working memory. During VTE, extended neuronal sweeps may support deliberative processes. Awake replay could also support trajectory planning, on-line consolidation and reconsolidation, as well as on-line retrieval of long-term memory. Thus, it remains currently unclear which specific functions are sustained by theta sequences, awake replay, or both.

Finally, a growing number of studies have provided evidence that hippocampal activity can also convey non-spatial information, including about objects, odors, sound sequences, time, goals, fear, etc. (e.g., Wood et al., 1999; Moita et al., 2003; Macdonald et al., 2011; Aronov et al., 2017). This suggests a general mechanism for encoding continuous, task-relevant variables. Recent findings indicate that phase precession may also represent a potential code for non-spatial features (Terada et al., 2017). Future work will be required to determine how non-spatial information is integrated at the fine timescale during hippocampal computation.

## Author Contributions

All authors listed have made a substantial, direct and intellectual contribution to the work, and approved it for publication.

### Conflict of Interest Statement

The authors declare that the research was conducted in the absence of any commercial or financial relationships that could be construed as a potential conflict of interest.

## Footnotes

^1^Incidentally, in its “uncorrected” version, this model predicted that place cells should fire in regular spatial patterns long before the discovery of grid cells (see e.g., Lengyel et al. (2003) for an influential model which eliminates repeated fields).

## Abbreviations

Cell assembly.: Group of neurons that transiently co-activate during a certain function or mental process. Place cells with highly similar fields can fire within a brief window of time (common theta phase, often grouped within a gamma cycle) and form a cell assembly. Reciprocal connectivity between group members may or may not be implied (e.g., cell assemblies in CA3 vs. CA1).
Theta sequence.: Sequential activation of hippocampal cell assemblies paced by the ongoing theta rhythm (~8 Hz) and representing the ongoing trajectory at a highly accelerated rate. For instance, when a rat successively traverses place fields A, B, and C (which lasts on the order of a second), in the overlapping region, ongoing theta cycles contain spikes A, B, and C in the same order (within only ~150 ms). Theta sequences typically span one theta cycle, but longer sequences have also been described.
Replay.: Reinstatement during offline states of the sequential activity observed during behavior. Replay is associated with sharp wave ripple complexes and can occur both in the forward or backward direction.
Theta phase precession.: The time at which a cell fires relative to the ongoing theta cycle (“phase”) decreases as the animal moves through space. Thus, the cell initially fires at the end of a theta cycle, then slightly before the end of the next cycle, until it eventually fires its last spikes near the beginning of the ongoing theta cycle. This phenomenon is called “phase precession.” Initially described in place cells, phase precession has also been reported in other hippocampal cells, including interneurons, “episode,” and sound-responsive cells (in which case the movement is considered in a more abstract space), as well as in other brain structures such as the medial prefrontal cortex or the ventral striatum.
Nested oscillations.: Intermingled oscillations of different frequencies, especially when the phase of the slower oscillation modulates the amplitude of the faster oscillation. For instance, slow, medium and fast gamma bursts tend to occur at different phases of theta (phase-amplitude modulation).
Nested sequences.: As a rat explores an environment, place cell assemblies activate in sequence and thus form “behavioral” (slow time scale) sequences representing the ongoing trajectory in real time. Simultaneously, the same assemblies form theta (fast time scale) sequences that describe the same trajectory, nested within the behavioral sequences.
